# Homemade pericardial bifurcated graft for Q fever-infected abdominal aortic aneurysm open repair: a case report

**DOI:** 10.3389/fcvm.2024.1418949

**Published:** 2024-05-28

**Authors:** A. Mouche, J. Baron, C. Toquet, T. Le Corvec, B. Maurel, A. Benichou, J. Mougin

**Affiliations:** ^1^ Vascular Surgery, Hospital G&R Laennec—CHU Nantes, St Herblain, France; ^2^ Pathology Department, CHU Nantes, Nantes University, Nantes, France; ^3^ L'Institut du Thorax, University of Nantes, CHU Nantes, CNRS, INSERM, Nantes, France; ^4^ Vascular Medicine, Hospital G&R Laennec—CHU NANTES, St Herblain, France

**Keywords:** Q fever, *Coxiella burnetii*, abdominal aortic aneurysm, inflammatory aortic disease, vascular surgery

## Abstract

Q fever is a zoonotic infection caused by *Coxiella burnetii*. In rare cases, it can lead to vascular complications, including infected aneurysms. Successful treatment involves surgery and antibiotics, but there is no established consensus or clear recommendation for the choice of material graft. We report a case of abdominal aortic aneurysm infected by *C. burnetii* treated by open surgery with complete resection of the aneurysm and homemade bovine pericardial bifurcated graft reconstruction and long-term antibiotherapy using doxycycline. One year postoperatively, the patient had no sign of persistent infection or vascular complication. Moreover, *C. burnetii* immunoglobulins titers decreased 6 months postoperatively.

## Introduction

*Coxiella burnetii* is a Gram-negative intracellular bacterium responsible for Q fever. This is a zoonotic infection with a wide range of hosts, including ruminants. Aerogenic transmission is the major source of dissemination but rare cases of Q fever transmission through tick bites or by crushing a tick have also been documented (1).

C. *burnetii* infection can lead to acute or chronic infection. Most acute infections are asymptomatic or limited with a mild influenza-like illness and usually result in spontaneous recovery (2). Nevertheless, C. *burnetii* can persist within host macrophages despite apparent treatment success. Chronic Q fever is marked by significantly higher mortality rates, reaching 25% (3), characterized by the emergence of valvular abnormalities, infected aortic aneurysms, or vascular prostheses (4), which, if left untreated, frequently result in fatal outcomes (5). The most common symptoms are non-specific, with abdominal pain, fatigue, and weight loss leading to a diagnostic delay. The serologic presentation is characterized by an increase in both phases of IgM and IgG antibodies (6).

The physiopathological mechanism responsible for *C. burnetii*-induced vascular damage involves a complex interplay between various factors. This includes chronic inflammation due to intracellular infection persistence, the host's immune responses, activation of endothelial cells lining the blood vessels, and processes related to thrombosis (7). A comprehensive understanding of these mechanisms is critical for the development of effective therapeutic strategies for Q fever.

Surgical treatment of Q fever-infected aneurysms is not well defined; in particular, the choice of material graft and the risk of recurrence are high, even after aggressive surgical therapy (8).

In this report, we present a case of chronic Q fever-infected abdominal aneurysm in a 73-year-old man with no prior vascular disease treated with complete resection of the aneurysm and homemade bovine pericardial bifurcated graft reconstruction.

## Case report

A 73-year-old man, with a medical history of non-ST segment elevation myocardial infarction treated by medical therapy, hemorrhagic rectocolitis diagnosed in 2002 under anti-TNF-α therapy in histological and endoscopic remission and epidermoid cyst infection, consulted for persistent inflammatory abdominal pain. The patient lived in a semi-rural area and did not report any specific contact with livestock.

### Diagnosis

Computed tomography angiography (CTA) showed an infra renal abdominal aortic aneurysm with a maximal diameter of 42 mm, with anterior parietal thickening and extension to the left common iliac artery.

An [^18^F]fluorodeoxyglucose-positron emission tomography/computed tomography (18FDG-PET/CT) scan revealed a significant uptake of aneurysm wall with no argument for large- or medium-vessel vasculitis.

The patient was evaluated for zoonotic infections. Immunofluorescence assay revealed significantly elevated *C. burnetii* immunoglobulin titers consistent with chronic Q fever: IgG phase I of 1:512, IgM of <1:32, and IgG phase II of 1:256. Moreover, there was no clinical or transthoracic echocardiographic argument for endocarditis.

### Medical treatment

**Figure 1:**
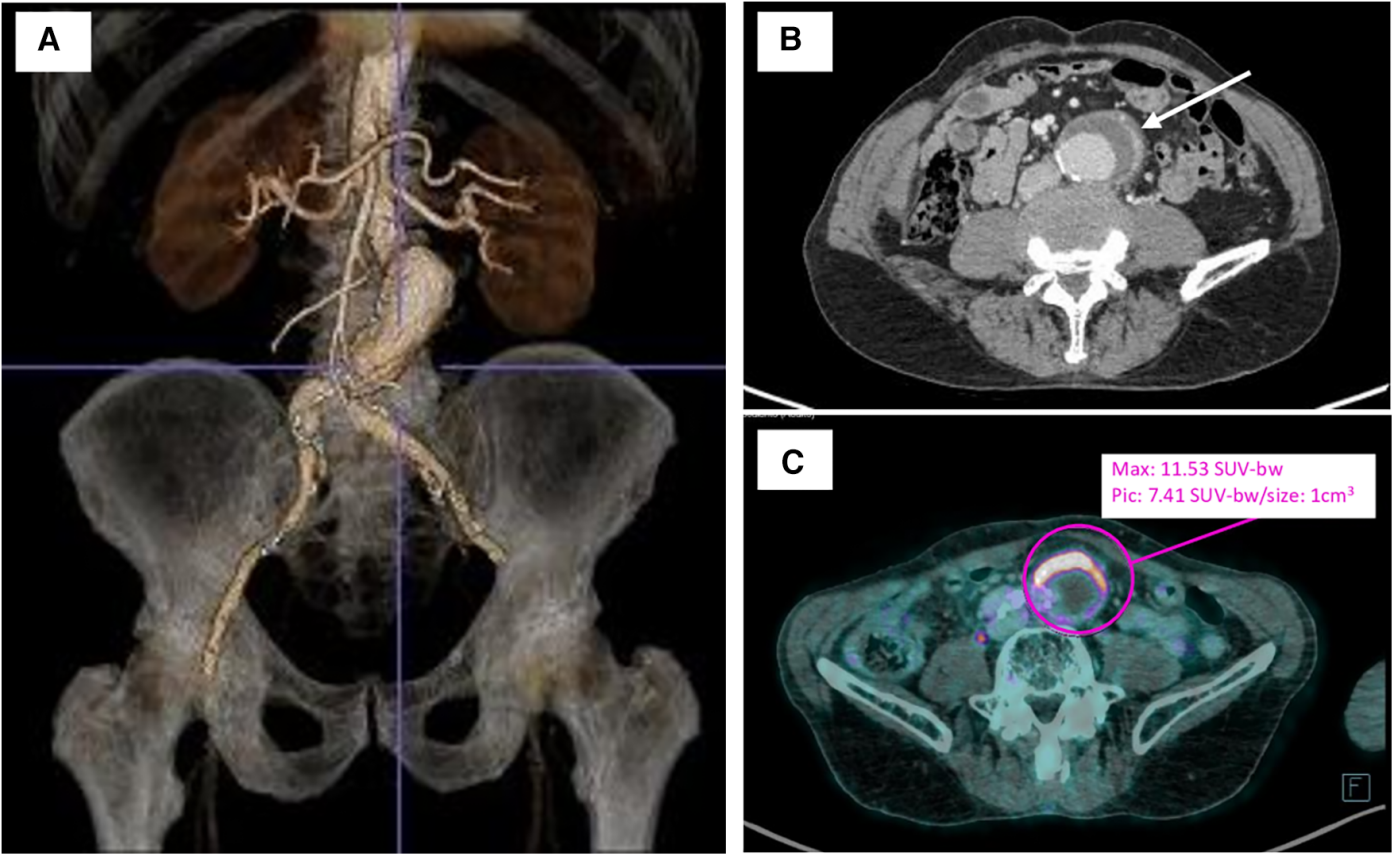
Preoperative imagery of infrarenal aortic aneurysm. (**A**) Three-dimensional computed tomography aortic aneurysm reconstruction with the TeraRecon Aquarius workstation preoperatively. (**B**) Preoperative computer tomography angiography of the infected abdominal aortic aneurysm with anterior aortic wall thickening and enhancement (white arrow). (**C**) Preoperative 18FDG-PET/CT revealing a significant uptake of anterior aneurysm wall (purple circle). bw, body weight.

Given the evidence of chronic Q fever infection, the patient was initially treated using doxycycline and hydroxychloroquine. Serologic control realized 3 weeks after the initiation of therapy showed a stability of phase I and II antibody titers. During the follow-up (FU), the patient described a persistence of inflammatory abdominal pain, and at the 6-month FU, CTA showed a significant progression of the aortic aneurysm (+6 mm) and an intense perianeurysmal contrast uptake indicative of ongoing inflammatory activity (Figure 1). After a multidisciplinary discussion, it was decided the patient would undergo open surgical repair with complete removal of the affected segment and surrounding tissue.

### Surgical technique

**Figure 2:**
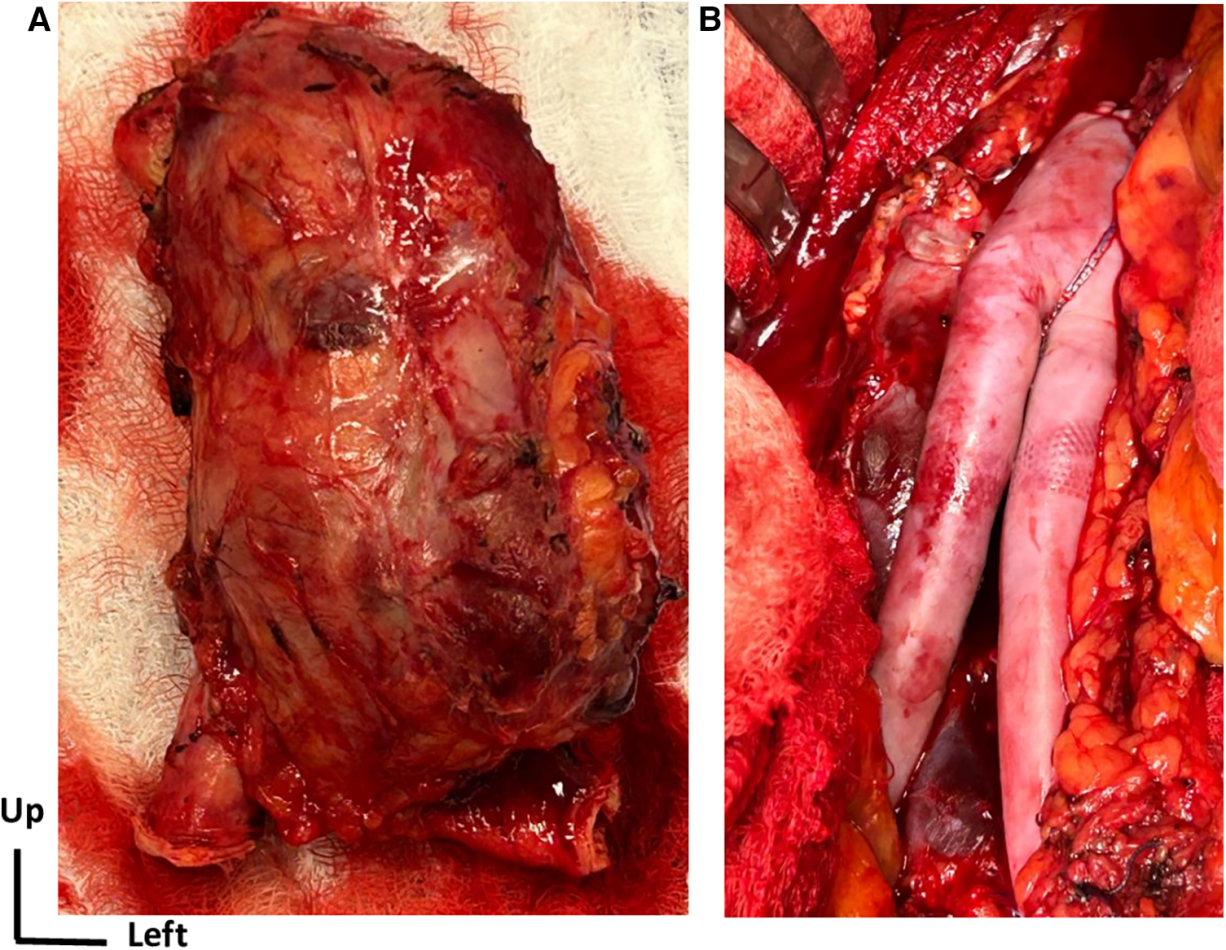
Surgical open repair treatment. (**A**) View of the complete removal of the affected aortic aneurysm segment along with the surrounding tissue. (**B**) Intraoperative view of aortic reconstruction using a self-made bifurcated graft with a bovine pericardial patch.

Under general anesthesia, laparotomy was performed, and the infrarenal aorta and distal common iliac arteries were clamped. The aneurysm was completely removed, and the aortic reconstruction used a self-tailored bovine pericardial bifurcated graft (Figure 2). This surgical technique has been previously described (9). The aneurysm was investigated for anatomopathological and bacteriological analysis. No early reintervention was required. The patient spent 1 day in the intensive care unit and was discharged from hospital after 7 days.

### Histological characteristics

**Figure 3:**
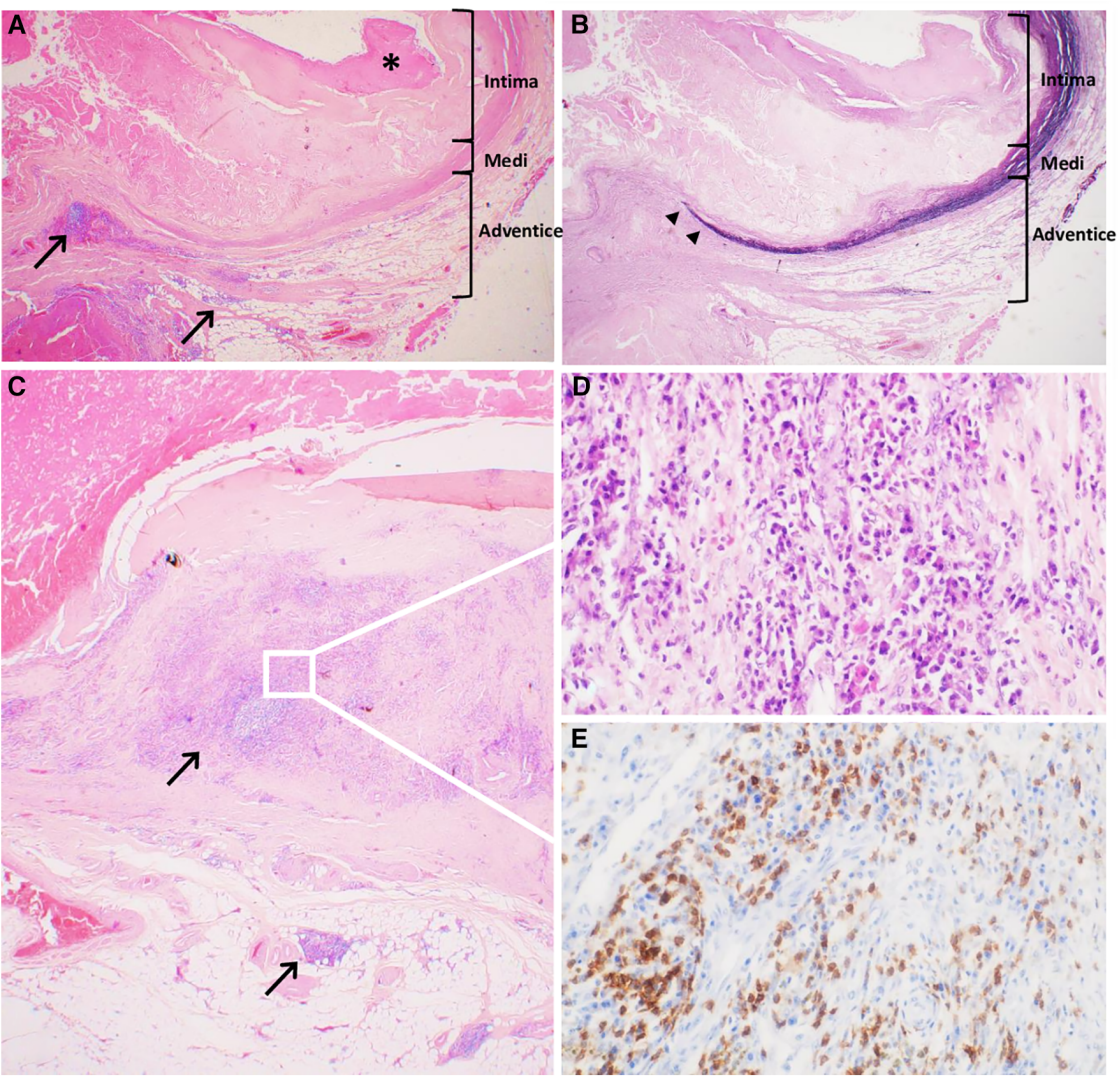
Historical characteristics of the abdominal inflammatory aneurysm. (**A**) Severely altered aneurysmal wall with complex atherosclerotic plaques, thickened intima and surface thrombus (*), fragmented medial layer, and dense connective tissue in the adventitial layer inflammatory lesions next to atherosclerotic lesions but also far away, indicated with black arrows (Hematoxyline-Eosine-Safran, original magnification ×20). (**B**) Severely altered aneurysmal wall with disrupted architecture with loss of elastin fibers in the medial layer (‣) (Weigert, original magnification ×20). (**C**) Aortitis with abundant inflammatory infiltrate, especially in the adventitial layer, composed of lymphocytes and plasma cells (Hematoxyline-Eosine-Safran, original magnification ×20). (**D**) Zoomed-in section demonstrated the lymphoplasmacytic pattern of the inflammatory infiltrate (Hematoxyline-Eosine-Safran, original magnification ×400). (**E**) Zoomed-in section shows strong membranous and cytoplasmic straining of lymphocytes of anti-CD3 antibody (Immuno Histo Chemistry, original magnification ×400).

Histologic examination of the aorta revealed a thickened and severely damaged aneurysmal wall. We noticed complex atherosclerotic plaques with thickened intima (by the fibrous tissue with lipid and calcific deposits and a fibrinocruoric thrombus partially incorporated into the wall); the underlying media was attenuated, fragmented, or replaced by fibrous tissue. The adventitia was replaced by a dense connective tissue sometimes almost in direct continuity with the thrombus. Furthermore, we observed aortitis characterized by an abundant inflammatory infiltrate, especially in the adventitial layer, which was consistent with the PET scan that revealed significant uptake of the aneurysm. The inflammatory infiltrate consisted of CD3+ CD8+ T lymphocytes, CD138+ plasma cells, CD68+ CD163+ macrophages, and rare giant cells without well-defined granuloma (Figure 3). According to the Consensus Statement on Surgical Pathology of the Aorta, this would correspond to the lymphoplasmacytic pattern. Polymerase chain reaction (PCR) detected C. *burnetii* on a perioperative aortic sample.

### Follow-up

Postoperatively, the patient was maintained on doxycycline and hydroxychloroquine for a minimum duration of 12 months, and this therapy is still on course. The CT scan performed 2 months postoperatively showed good patency of the graft with no early argument for scanographic recurrence of Q fever infection.

However, *C. burnetii* immunoglobulin titers remained stable without a significant decrease at 4 months postoperatively. Finally, *C. burnetii* immunoglobulin titers decreased after 6 months postoperatively (IgG phase I of 1:256 vs. 1:512).

## Discussion

Chronic Q fever is a rare but life-threatening condition. Infected arterial aneurysms are one of the most frequent complications (14%) with endocarditis (13%) in patients with proven chronic Q fever and are associated with a high risk of mortality (up to 38%) (10). The diagnosis of intracellular bacterial infections remains difficult for several reasons: unspecific and varied symptomatology and non-detection by conventional culture methods, and their identification requires special tests that must be specifically requested by the clinician.

In this case, the patient had no obvious history of *C. burnetii* exposure but exhibited two factors predisposing to chronic Q fever: a history of immunodepression (anti-TNF-α therapy since 2002) and probably a pre-existing atherosclerotic aortic aneurysm before the *C. burnetii* infection.

TNF-α plays a crucial role in the host defense against intracellular pathogens. The inhibition of TNF-α can impair the immune response and increase susceptibility to infections, including the reactivation of latent infections or acquisition of new ones (11).

Several case reports and epidemiological studies have documented cases of Q fever in patients receiving anti-TNF-α therapy with atypical or severe forms of Q fever, including chronic Q fever, endocarditis, or disseminated disease (12, 13).

Moreover, perioperative histological analysis revealed significant atherosclerosis lesions with ulcerated plaques testifying the probable anterior presence of an atheromatous aneurysm. Atherosclerosis is known as a facilitating factor of *C. burnetii* aortitis development. Indeed, thrombi and atherosclerotic plaques are considered to be immunodeficient sites that enable bacteria growth.

The role of surgery appears as a crucial component in the management of aortic infections caused by *C. burnetii*. Complete excision of the aortic infected aneurysm is required to achieve total resolution of the infection (14), in association with recommended antibiotic therapy (15). Endovascular therapy should be reserved for patients who are unable to undergo open surgery.

The optimal graft substitute for an infected aortic aneurysm remains unclear and, more specifically, for a Q fever-infected aneurysm where the risk of recurrence is high despite aggressive surgical treatment. The European Society for Vascular Surgery (ESVS) guidelines (17) recommend repair with deep femoral veins, cryopreserved allografts, silver grafts, or rifampicin impregnated grafts (16). In our case, we decided to perform the aortic reconstruction using a homemade pericardial bifurcated graft to avoid additional extensive harvesting trauma using deep femoral veins. An appropriately sized cryopreserved cadaveric arterial allograft was not available for this case, and rifampicin-coated polyester grafts, which could have been easily used, remain associated with a high risk of reintervention (18); in addition, rifampicin is not very effective at treating *C. Burnetii* infection (19). Off-the-shelf pericardial patches can be easily stored for urgent cases. A homemade bifurcated graft preparation takes no more time than harvesting femoral veins or thawing and preparing cryopreserved allograft. No study has yet assessed the benefit of a homemade pericardial aortic graft for Q fever infection specifically, but its use seems to be associated with good clinical results in the mid-term with no clinical reinfections or graft-related complications for the treatment of native and aortic graft or endograft for all types of infection (20).

## Conclusion

Infected aortic aneurysm is a rare but life-threatening complication in patients with proven Q fever infection. Aggressive surgery with complete resection of the aneurysm associated with antibiotics is mandatory to achieve complete healing. A homemade bifurcated graft reconstruction using off-the-shelf bovine pericardial patch appears to be a feasible and safe alternative to autologous vein reconstruction and cryopreserved allografts.

## Data Availability

The raw data supporting the conclusions of this article will be made available by the authors, without undue reservation.
